# Myelomeningocele Surgery over the 10 Years Following the MOMS Trial: A Systematic Review of Outcomes in Prenatal versus Postnatal Surgical Repair

**DOI:** 10.3390/medicina57070707

**Published:** 2021-07-12

**Authors:** Francesca Gabriela Paslaru, Anca Maria Panaitescu, George Iancu, Alina Veduta, Nicolae Gica, Alexandru Catalin Paslaru, Anamaria Gheorghiu, Gheorghe Peltecu, Radu Mircea Gorgan

**Affiliations:** 1 Neurosurgical Department, “Bagdasar-Arseni” Clinical Emergency Hospital, 041915 Bucharest, Romania; francesca.paslaru@gmail.com (F.G.P.); ana.gheorgiu@gmail.com (A.G.); radu.gorgan@umfcd.ro (R.M.G.); 2 Neurosurgery Department, “Carol Davila” University of Medicine and Pharmacy, 020021 Bucharest, Romania; 3 Filantropia Clinical Hospital, 11171 Bucharest, Romania; george.iancu@umfcd.ro (G.I.); alina.veduta@gmail.com (A.V.); gica.nicolae@umfcd.ro (N.G.); gheorghe.peltecu@umfcd.ro (G.P.); 4 Obstetrics and Gynecology Department, “Carol Davila” University of Medicine and Pharmacy, 020021 Bucharest, Romania; 5 Physiology Department, “Carol Davila” University of Medicine and Pharmacy, 020021 Bucharest, Romania; catalin.paslaru@umfcd.ro

**Keywords:** spina bifida, neurosurgery, fetal surgery

## Abstract

*Background and Objectives*: Myelomeningocele is the most severe form of spina bifida, a congenital neural tube defect arising from an incomplete neural tube closure during early development with damage worsening with advancing gestational age. The Management of Myelomeningocele Study (MOMS) Trial proved that surgery performed before 26 weeks of gestation significantly improved the prognosis, significantly changing treatment paradigms. This article aims to provide a review of the changes and updates in spina bifida repair over the 10-year period following the MOMS Trial. *Material and methods:* We performed a systematic review in the PubMed and Cochrane databases as well as a hand-search of high-impact journals using the reference list of all identified articles, searching for randomized controlled trials and observational studies. *Results:* We identified 27 articles published between 2011 and 2021 that fulfilled the inclusion criteria and review them in the present study. *Conclusions:* With growing experience and with the improvement of prenatal open and fetoscopic techniques, the outcome of SB-associated conditions could be improved and the risks to both the mother and the fetus reduced. A continuous follow-up of the treated infants and further randomized trials are essential to study the complications and advantages or disadvantages of any given treatment strategy.

## 1. Introduction

Myelomeningocele (MMC), the most severe form of spina bifida (SB), is a congenital neural tube defect occurring with an incidence of approximately 1 in 2900 live births, arising during early development from an incomplete neural tube closure leading to an open spinal canal. The exposed spinal cord undergoes significant damage early in pregnancy due to the intrauterine environment with the spinal injury extending cranially and worsening with advancing gestational age [1,2], followed by a suction gradient on the hindbrain [3]. These events account for the basis of the “two-hit” hypothesis, the rationale behind prenatal surgery for an early MMC closure. The Management of Myelomeningocele Study (MOMS) Trial published in 2011 proved that surgery performed before 26 weeks of gestation significantly improved the prognosis by decreasing the risk of death or need for shunting by 12 months of age, reducing the degree of hindbrain herniation associated with Chiari II malformation (CM-II) and improving motor function and the likelihood of independent walking compared with postnatal surgery [4], thus significantly changing the treatment paradigms. This article aims to provide a review of the changes and updates in spina bifida repair over the 10-year period following the MOMS trial and to discuss the evidence of the superiority of prenatal versus postnatal surgical treatment in regard to both short-term and long-term prognoses.

## 2. Materials and Methods

We performed a systematic review in the PubMed and Cochrane databases as well as a hand-search of high-impact journals using the reference list of all identified articles, searching for randomized controlled trials and observational studies. The terms used for the search were “spinal dysraphism”, “spina bifida” or “myelomeningocele” combined with “surgery” or “f(o)etoscopy”. To be eligible for inclusion, studies had to be published between 2011 and 2021, written in English and reporting singleton fetuses with isolated spina bifida who underwent either a prenatal fetoscopic or open repair or postnatal surgical closure with a minimum of 30 days follow-up. Exclusion criteria were unrelated, duplicated, unavailable full-text or abstract-only papers. The primary outcome was perinatal mortality i.e., deaths during the fetal and postnatal (within the first month of life) periods. The secondary outcomes were maternal, fetal, neonatal and infant outcomes. The articles were categorized as either early experience or later experience, using a cutoff of 30 cases as proposed by Kohl et al. [5]. Nonrandomized studies were assessed for the risk of bias using the Newcastle–Ottawa Scale and judged on three perspectives: the selection of the study groups, the comparability of the groups and the outcome of interest [6]. The statistical significance was determined using the chi-squared and t-test statistics; *p* values < 0.05 were considered statistically significant.

## 3. Results

### 3.1. Description of Studies

**Figure 1:**
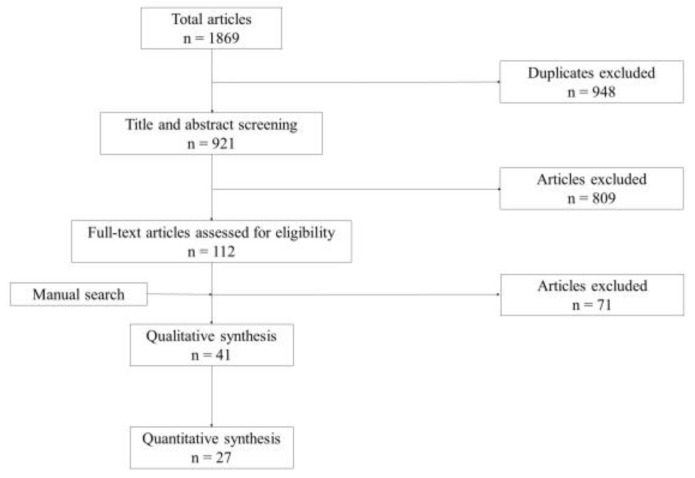
Study flow diagram adapted from the PRISMA flow diagram [7].

Our preliminary literature search identified 1869 publications; we used the PRISMA algorithm to assess them [7]. Duplicates were excluded, leaving us with 921 articles out of which 809 were excluded on reading the title and abstract. The remaining 112 were assessed for eligibility by full-text reading. The selection left us with 27 eligible articles that were included in the study (Figure 1) [8,9,10,11,12,13,14,15,16,17,18,19,20,21,22,23,24,25,26,27,28,29,30,31,32,33,34].

The articles were categorized as either early experience or later experience, using a cutoff of 30 cases as proposed by Kohl et al. [5] (Table 1). In the Table, we have listed the articles in order based on the year of publication starting from the oldest to the latest.

### 3.2. Risk of Bias

The 27 nonrandomized studies were assessed by two independent observers (FGP and ACP) for the risk of bias using the Newcastle–Ottawa Quality Assessment Scale (NOS) Cohort Studies and judged on the following three perspectives: 1. the selection of the study groups: the representativeness of the exposed cohort, the selection of the nonexposed cohort, the ascertainment of exposure and the demonstration that the outcome of interest was not present at start of the study; 2. the comparability of cohorts on the basis of the design or analysis and 3. outcome: the assessment of outcome, a follow-up long enough for outcomes to occur and the adequacy of follow-up cohorts [6] (Table 1). The differences between the observers were solved by consensus.

### 3.3. Operative, Maternal, Fetal, Neonatal and Infant Outcomes

Table 2 summarizes the maternal outcomes after prenatal interventions as reported in different studies. Two studies with early experience (defined as less than 30 patients) reported placental abruption rates of 10% [28] and 0% [23] whereas studies including more than 30 patients reported an average rate of placental abruption of 5.9% [17,19,24,25]. Only two studies, both having late experience, reported pulmonary edema rates of 2% [24] and 2.98% [17], respectively. Two series, both including more than 30 patients, reported chorioamnionitis rates of 4% [24] and 2.95% [25]. PPROM seemed to be less frequent in late experience versus early experience studies (*p* < 0.01) [13,17,19,23,24,25,28]. Comparing fetoscopic and open techniques, an average rate of placental abruption of 9.41% was reported in fetoscopic series [19,28] and of 3.69% in series using open techniques, respectively. The average rate of PPROM in series using fetoscopic techniques was 63.23% [17,28] and 30.71% in open surgery series [17,23,24,25].

Table 3 summarizes the operative and delivery outcomes in series reporting prenatal interventions. The average uterine dehiscence rate was 0.79% in early experience groups [17,19,25] and 0% in late experience series [28]. Three open surgery and late experience series reported hemorrhage rates with an average of 4.51% [17,24,25]. The mean gestational age at delivery appeared to be higher in late experience series compared with early experience ones (*p* < 0.01) [10,12,13,14,19,23,24,25,27,28].

The fetal, neonatal and infant outcomes are summarized in Table 4 and Table 5. The average hydrocephalus rates were 45.65% in prenatal series [10,12,14,23,24,28,33,34] and 66.57% in those reporting a postnatal treatment [8,9,11,15,16,18,22,26,29,30,31]. The ability to walk seemed to be slightly higher in prenatal series (68.78%) [10,34] than in postnatal groups (60.24%) [8,16,22,30,31,32]. The urinary continence average rate was also higher in prenatal series (40.97%) [20,27,28], compared with postnatal studies (8.94%) [15,27,29,30]. Additional SBA recoverage average rates were 11.9% in prenatal series [14,23,25] and 7.93% in the postnatal groups [11,16]. Only one prenatal study reported a retethering rate of 2.81% [14] whereas the average retethering rate in postnatal studies was 11% [8,15,18,29,31,32]. A reversal of the hindbrain herniation rates was higher in prenatal (63.14%) [12,13,23,24,25,28,33] than in postnatal groups (33.71%) [8,9,13,15,16,23,31].

Comparing fetoscopic and open techniques in prenatal treatment series, we noticed a higher average hydrocephalus rate in fetoscopic surgery groups (54.98%) [14,28,33,34] than in open surgery groups (36.31%) [10,12,23,24]. Only one fetoscopic treatment group reported the ability to walk with a rate of 54.23% [34] and one open treatment group reported an ability to walk rate of 33.33% [27]. The urinary continence rate was 71.42% in one fetoscopic surgery group [28] and 33.33% in one open surgery group [27]. Additional SBA recoverage was needed in 28.16% in one fetoscopic surgery study [14] compared with 2.54% [25] and 5% [23] in open surgery series. A hindbrain herniation reversal was achieved in 93.85% in fetoscopic surgery series [28,33] and in 51.25% in open surgery series [12,13,23,24,25].

Comparing early to late experience series, the hydrocephalus rate was slightly higher (49.29%) in early experience groups [10,23,28,33] than that reported in late experience series (42%) [12,14,24,34]. Two early experience studies reported urinary continence rates of 18.18% [20] and 71.42% [28] and one late experience study reported a rate of 33.33% [27]. Additional SBA recoverage was needed in 5% of cases in an early experience study [23] versus 2.54% [25] and 28.16% [14] in late experience groups. A hindbrain herniation reversal was quite similar in early versus late experience series of 63.57% and 62.82%, respectively [12,13,23,24,25,28,33].

### 3.4. Mortality Rate

The comparison between infant mortality rates in different neurosurgery centers is summarized in Table 6. The mortality rate in prenatal series was not high (between 2% and 20%). Many postnatal series reported no immediate infant mortality.

### 3.5. Comparison between Surgical Techniques

The comparison between surgical techniques in different centers is summarized in Table 7. Out of the 25 articles that reported a single surgical technique, 12 (48%) reported a postnatal closure of the defect, 6 (27.3%) reported open prenatal surgery, 5 (20%) reported fetoscopic prenatal surgery and 2 (9%) reported a prenatal surgical intervention not otherwise specified. Pastuszka et al. [27] and Flanders et al. [13] reported both open prenatal and postnatal surgical interventions.

### 3.6. Comparison between the Results of the Reported Approaches

The studies included reported on many different outcomes. For a few of these outcomes, a comparison between the results obtained in specific management scenarios (prenatal versus postnatal treatment, early experience versus late experience) is possible. Overall, mortality was higher in prenatally-treated than in postnatally-treated cases (*p* < 0.01). Hydrocephalus and Chiari malformations were more frequent in the postnatally-treated than in the prenatally-treated cases (*p* < 0.05 for both outcomes). The rupture of membranes was less frequent and the gestational age at delivery was higher in late experience versus early experience series (*p* < 0.01 for both). Many outcomes were reported only by a few studies. The follow-up interval was different in different studies.

## 4. Discussion

A large amount of heterogenous data has lately been published on the in utero repair of spina bifida. As these data on fetal surgery accumulate, the results are judged against those of the conventional postnatal surgery for spina bifida. This analysis does not seem to be close to any conclusions and the approach that would benefit patients most is still not clearly known. Systematic reviews and meta-analyses are useful tools for drawing practical conclusions and making sense of heterogenous literature.

The history of surgical interventions for myelomeningocele starts with a series of trials and errors from a time when the rules regarding who and when should operate were not clearly defined. Many strategies have been tried over the years such as ligatures, injections, serial tapping and excision. While these early trials proved ineffective, they served to build the knowledge of the pathology involved and eventually resulted in modern approaches [35].

Miled et al. studied the timewise progression and topographic progression of neuronal loss in 186 cases of myelomeningocele and reported that a significant neuronal loss is present earlier than a gestational age of 16 weeks and progressively extends cranially, thus suggesting that an earlier prenatal repair could prevent Chiari II malformation, rescue the remaining motor neurons in the exposed cord and prevent the extension to the upper levels of the spinal cord [2]. Diagnosing fetal myelomeningocele can be done during routine antenatal appointments. Munoz et al. compared a prenatal ultrasound evaluation to a prenatal MRI and stated that MRI is not superior to ultrasound in the diagnosis of open spina bifida [36]. Recent advances in ultrasound technology allow for an early diagnosis at 12–14 weeks’ gestation of severe spina bifida [37]. Spina bifida is sometimes associated with genetic anomalies such as trisomy 18. Both major structural defects and common aneuploidies can be routinely diagnosed in the first trimester of pregnancy [38,39].

The MOMS Trial, published in 2011 in the *New England Journal of Medicine*, was the largest randomized trial on the prenatal versus the postnatal treatment of myelomeningocele and aimed to confirm the benefits of in utero surgical interventions earlier than 26 gestational weeks and to assess the risks of such interventions [4]. According to the MOMS Trial, prenatal surgery for myelomeningocele significantly reduced the need for a cerebrospinal fluid shunt and significantly improved motor function at 30 months but came with costs in terms of both maternal and fetal morbidity. The inclusion criteria were a maternal age of over 18 years, US residency, a singleton pregnancy, an upper myelomeningocele boundary between T1 and S1, a hindbrain herniation, a gestational age between 19.0 and 25.9 weeks and a normal karyotype. The exclusion criteria were either regarding the mother such as a contraindication to surgery (e.g., a previous hysterotomy), a body mass index over 35 and a risk of preterm birth and placental abruption or regarding the fetus such as severe kyphosis or a fetal anomaly unrelated to myelomeningocele [4].

Antiel et al. studied the impact on family and parental stress of prenatal and postnatal repairs of myelomeningocele and reported that the overall negative parental impact of caring for a child with myelomeningocele was significantly lower in the antenatal treatment group compared with the postnatal treatment group, with the ambulation status and family resources being predictive of the impact on parental and family stress [40].

The results of the MOMS trial are clearly encouraging [4,41] but historically they have to be considered as early experience with fetal surgery. Research on the in utero repair of spina bifida continues and several fetal medicine centers have published their ‘post-MOMS’ experience on fetal surgery for spina bifida [8,9,10,11,12,13,14,15,16,17,18,19,20,21,22,23,24,25,26,27,28,29,30,31,32,33,34]. More importantly, the postnatal repair of spina bifida carries on, for several obvious reasons, in many pediatric neurosurgery centers and yields good results [42]. In our study, 50% of the articles still reported a postnatal closure of the defect in cases of myelomeningocele. One of the latest studies included in our analysis [32] challenged the idea that the results of a prenatal repair of SB are significantly better than those of a postnatal repair. The study showed that the tethering of the spinal cord occurs at a higher rate in prenatally-treated patients than in postnatally-treated patients [32].

In the post-MOMS era, the therapeutic landscape has become more complex with the emergence of new (fetoscopic) techniques and approaches for in utero surgery for spina bifida.

Joyeux et al. in 2015 [43] and Kabagambe et al. in 2018 [44] compared a fetoscopic and an open repair of myelomeningocele and stated that the newer fetoscopic method takes longer to complete, has a greater risk of prematurity and requires additional postnatal procedures but has a comparable shunt rate and is not linked to uterine thinning or dehiscence [43,44]. However, larger studies and long-term data are needed.

Reviews of the literature on spina bifida management have been published over the entire decade since the MOMS trial [45,46,47]. A consensus statement of the Congress of Neurological Surgeons was based on the literature up to 2016 and was very much influenced by the initial results of the MOMS trial [45]. The methodology of systematic reviews varies widely and is sometimes overly stringent. For example, a meta-analysis from 2019 included only two studies out of all available literature: the 30-month results of the MOMS trial and a prospective cohort published in 2014 [47]. In this context, we consider our review not superfluous.

The 27 studies included in our review heterogeneously report on many different outcomes. Unfortunately, many outcomes were reported only by a few studies with sometimes conflicting results. For instance, of the two studies containing data on the gestational age at delivery in fetoscopic prenatal surgery, one reported an extremely low (32 weeks) mean gestational age at delivery [28] whereas the other reported an extremely high (38 weeks) mean gestational age at delivery [19]. The follow-up interval varied widely among the studies; therefore, a meaningful analysis of the results such as the ability to walk or retethering is difficult. We feel that a substantial statistical analysis of the prognosis associated with different treatment strategies is not possible because of the lack of uniformity of data reporting. Bias can be introduced by the retrospective design of the majority of studies. Interpretations of the data should be cautious.

## 5. Conclusions

Although there have been 10 years since the MOMS Trial, experience still needs to be gained and prenatal techniques need to be improved in order to obtain a better prognosis and lower risks for both the mother and fetus. Despite the apparent benefits of fetal surgery, only a few centers offer this technique, which is more technically demanding and requires a multidisciplinary effort [48]. With growing experience and with the improvement of prenatal open and fetoscopic techniques, the outcome of SB-associated conditions could be improved and the risks to both the mother and the fetus reduced. A continuous follow-up of the treated infants and further randomized trials with a uniform design are essential to study the complications and advantages or disadvantages of any given treatment strategy.

## Figures and Tables

**Table 1:** Study assessment of quality using the Newcastle–Ottawa scale [6] and based on experience.

Author, Year of Publication	Cases (n)	Experience	NOS		
			Selection	Comparability	Outcome
Haq et al., 2012 [15]	25	Early	***	*	**
Lee et al., 2012 [20]	11	Early	****	*	***
Musluman et al., 2012 [26]	162	Late	***	**	**
Faria et al., 2013 [10]	6	Early	****	**	***
Macedo et al., 2015 [21]	19	Early	***	*	***
Moldenhauer et al., 2015 [24]	100	Late	****	**	**
Cherian et al., 2016 [11]	114	Late	****	*	**
Graf et al., 2016 [14]	71	Late	***	**	***
Januschek et al., 2016 [16]	48	Late	***	**	**
Pedreira et al., 2016 [28]	10	Early	***	**	***
Beuriat et al., 2017 [9]	61	Late	***	*	***
Elbabaa et al., 2017 [12]	55	Late	***	**	**
Kahr et al., 2018 [17]	67	Late	****	**	**
Kellogg et al., 2018 [18]	153	Late	***	**	***
Kohn et al., 2018 [19]	34	Late	***	*	**
Moron et al., 2018 [25]	237	Late	***	**	**
Pastuszka et al., 2018 [27]	36 prenatal/ 36 postnatal	Late	****	*	***
Beuriat et al., 2019 [8]	29	Early	***	*	***
Carraba et al., 2019 [33]	5	Early	***	*	**
Masini et al., 2019 [22]	157	Late	****	**	***
Mohrlen et al., 2019 [23]	20	Early	***	**	***
Protzenko et al., 2019 [29]	231	Late	***	**	***
Sileo et al., 2019 [30]	67	Late	***	**	***
Spoor et al., 2019 [31]	93	Late	***	**	***
Flanders et al., 2020 [13]	119 prenatal/62 postnatal	Late	****	**	***
Lapa et al., 2021 [34]	103	Late	***	**	***
Weaver et al., 2021 [32]	163	Late	***	**	***

*, **, ***, **** according to the NOS scale [6].

**Table 2:** Maternal outcomes in centers reporting prenatal surgical interventions.

Author, Year of Publication	Cases	Approach	Placental Abruption	Pulmonary Edema	Chorioamnionitis	PPROM
Faria et al., 2013 [10]	6	open	n.s.	n.s.	n.s.	n.s.
Moldenhauer et al., 2015 [24]	100	open	2 (2%)	2 (2%)	4 (4%)	31 (32.29%)
Graf et al., 2016 [14]	71	fetoscopic	n.s.	n.s.	n.s.	n.s.
Pedreira et al., 2016 [28]	10	fetoscopic	1 (10%)	0	0	10 (100%)
Elbabaa et al., 2017 [12]	55	open	n.s.	n.s.	n.s.	n.s.
Kahr et al., 2018 [17]	67	open	8 (11.94%)	2 (2.98%)	n.s.	19 (28.35%)
Kohn et al., 2018 [19]	34	fetoscopic	3 (8.82%)	n.s.	0	9 (26.47%)
Moron et al., 2018 [25]	237	open	2 (0.84%)	n.s.	7 (2.95%)	63 (26.58%)
Pastuszka et al., 2018 [27]	36	open	n.s.	n.s.	n.s.	n.s.
Mohrlen et al., 2019 [23]	20	open	0	0	0	7 (35%)
Flanders et al., 2020 [13]	119	open	n.s.	n.s.	n.s.	4 (3.36%)

n.s.—not stated.

**Table 3:** Operative and delivery outcomes in centers reporting prenatal surgical interventions.

Author, Year of Publication	Cases	Approach	Hemorrhage	Uterine Dehiscence	Mean Gestational Age at Delivery
Faria et al., 2013 [10]	6	open	n.s.	n.s.	32 weeks
Moldenhauer et al., 2015 [24]	100	open	1 (1%)	n.s.	34 weeks
Graf et al., 2016 [14]	71	fetoscopic	n.s.	n.s.	n.s.
Pedreira et al., 2016 [28]	10	fetoscopic	0	0	32 weeks
Elbabaa et al., 2017 [12]	55	open	n.s.	n.s.	34 weeks
Kahr et al., 2018 [17]	67	open	7 (10.44%)	1 (1.49%)	36 weeks
Kohn et al., 2018 [19]	34	fetoscopic	0	0	38 weeks
Moron et al., 2018 [25]	237	open	5 (2.1%)	2 (0.84%)	33 weeks
Pastuszka et al., 2018 [27]	36	open	n.s.	n.s.	n.s.
Mohrlen et al., 2019 [23]	20	open	0	2 (10%)	35 weeks
Flanders et al., 2020 [13]	119	open	n.s.	n.s.	34 weeks

n.s.—not stated.

**Table 4:** Operative fetal, neonatal and infant outcomes.

Author, Year of Publication	Patients	Approach	Additional SBA Recoverage	Retethering	Absence of CM-II
Haq et al., 2012 [15]	25	postnatal	n.s.	3 (12%)	1 (4%)
Lee et al., 2012 [20]	11	Prenatal—n.s.	n.s.	n.s.	n.s.
Musluman et al., 2012 [26]	162	postnatal	n.s.	n.s.	n.s.
Faria et al., 2013 [10]	6	prenatal—open	n.s.	n.s.	n.s.
Macedo et al., 2015 [21]	19	prenatal—n.s.	n.s.	n.s.	n.s.
Moldenhauer et al., 2015 [24]	100	prenatal—open	n.s.	n.s.	71.1%
Cherian et al., 2016 [11]	114	postnatal	4 (3.36)	n.s.	n.s.
Graf et al., 2016 [14]	71	prenatal—fetoscopic	20 (28.16%)	2 (2.81%)	n.s.
Januschek et al., 2016 [16]	48	postnatal	6 (12.5%)	n.s.	12 (25%)
Pedreira et al., 2016 [28]	10	prenatal—fetoscopic	n.s.	n.s.	6/7 (85.71%)
Beuriat et al., 2017 [9]	61	postnatal	n.s.	n.s.	33 (54.09%)
Elbabaa et al., 2017 [12]	55	prenatal—open	n.s.	n.s.	15 (27.27%)
Kellogg et al., 2018 [18]	153	postnatal	n.s.	24 (15.68%)	n.s.
Moron et al., 2018 [25]	237	prenatal—open	6 (2.54%)	n.s.	169 (71.4%)
Pastuszka et al., 2018 [27]	36	prenatal—open	n.s.	n.s.	n.s.
	36	postnatal	n.s.	n.s.	n.s.
Beuriat et al., 2019 [8]	29	postnatal	n.s.	4 (13.79%)	24 (82.75%)
Carraba et al., 2019 [33]	5	prenatal—fetoscopic	n.s.	n.s.	5 (100%)
Masini et al., 2019 [22]	157	postnatal	n.s.	n.s.	n.s.
Mohrlen et al., 2019 [23]	20	prenatal—open	1 (5%)	0	1 (5%)
Protzenko et al., 2019 [29]	231	postnatal	n.s.	11 (4.76%)	n.s.
Sileo et al., 2019 [30]	67	postnatal	n.s.	n.s.	n.s.
Spoor et al., 2019 [31]	93	postnatal	n.s.	11 (11.82%)	10%
Flanders et al., 2020 [13]	119	prenatal—open	n.s.	n.s.	97 (81.51%)
	62	postnatal	n.s.	n.s.	31 (50%)
Lapa et al., 2021 [34]	170	prenatal—fetoscopic	n.s.	n.s.	n.s.
Weaver et al., 2021 [32]	163	postnatal	n.s.	8%	10.40%

n.s.—not stated.

**Table 5:** Longer-term infant outcomes.

Author, Year of Publication	*N*	Approach	Hydrocephalus Treatment	Ability to Walk	Urinary Continence	Major Urological Surgery
Haq et al., 2012 [15]	25	postnatal	19 (76%)	n.s.	3 (12%)	n.s.
Lee et al., 2012 [20]	11	prenatal—n.s.	n.s.	n.s.	2 (18.18%)	2 (18.18%)
Musluman et al., 2012 [26]	162	postnatal	101 (62.34%)	n.s.	n.s.	n.s.
Faria et al., 2013 [10]	6	prenatal—open	2 (33.33%)	5 (83.33%)	n.s.	n.s.
Macedo et al., 2015 [21]	19	prenatal—n.s.	n.s.	n.s.	n.s.	1 (5.26%)
Moldenhauer et al., 2015 [24]	100	prenatal—open	2/83 (2.4%)	n.s.	n.s.	n.s.
Cherian et al., 2016 [11]	114	postnatal	26 (21.84%)	n.s.	n.s.	n.s.
Graf et al., 2016 [14]	71	prenatal—fetoscopic	32 (45.07%)	n.s.	n.s.	n.s.
Januschek et al., 2016 [16]	48	postnatal	41 (85.41%)	18 (37.5%)	n.s.	n.s.
Pedreira et al., 2016 [28]	10	prenatal—fetoscopic	3/7 (42.85%)	n.s.	5/7 (71.42%)	n.s.
Beuriat et al., 2017 [9]	61	postnatal	33 (54.09%)	n.s.	n.s.	n.s.
Elbabaa et al., 2017 [12]	55	prenatal—open	30 (54.54%)	n.s.	n.s.	n.s.
Kellogg et al., 2018 [18]	153	postnatal	137 (89.54%)	n.s.	n.s.	n.s.
Moron et al., 2018 [25]	237	prenatal—open	n.s.	n.s.	n.s.	n.s.
Pastuszka et al., 2018 [27]	36	prenatal—open	n.s.	n.s.	12 (33.33%)	n.s.
	36	postnatal	n.s.	n.s.	1 (2.77%)	n.s.
Beuriat et al., 2019 [8]	29	postnatal	11 (37.93%)	26 (89.65%)	n.s.	n.s.
Carraba et al., 2019 [33]	5	prenatal—fetoscopic	3 (60%)	n.s.	n.s.	n.s.
Masini et al., 2019 [22]	157	postnatal	115 (73.24%)	68/136 (50%)	n.s.	n.s.
Mohrlen et al., 2019 [23]	20	prenatal—open	11 (55%)	n.s.	n.s.	n.s.
Protzenko et al., 2019 [29]	231	postnatal	193 (83.54%)	n.s.	2 (0.86%)	n.s.
Sileo et al., 2019 [30]	67	postnatal	43 (64.17%)	13/53 (24.52%)	6/55 (10.9%)	n.s.
Spoor et al., 2019 [31]	93	postnatal	78 (83.87%)	18.60%	68 (74.73%)	n.s.
Flanders et al., 2020 [13]	119	prenatal—open	46 (38.65%)	n.s.	n.s.	n.s.
	62	postnatal	50 (80.64%)	n.s.	n.s.	n.s.
Lapa et al., 2021 [34]	170	prenatal—fetoscopic	68/103 (66.01%)	32/59 (54.23%)	36/59 (61.01%)	n.s.
Weaver et al., 2021 [32]	163	postnatal	n.s.	66%	n.s.	n.s.

n.s.—not stated.

**Table 6:** Infant mortality rates.

Author, Year of Publication	Patients	Approach	Infant Mortality
Beuriat et al., 2019 [8]	29	postnatal	0
Beuriat et al., 2017 [9]	61	postnatal	0
Faria et al., 2013 [10]	6	prenatal—open	0
Cherian et al., 2016 [11]	114	postnatal	2 (1.75%)
Elbabaa et al., 2017 [12]	58	prenatal—open	2 (3.44%)
Flanders et al., 2020 [13]	119	prenatal—open	10 (8.4%)
	62	postnatal	2 (3.22%)
Graf et al., 2016 [14]	71	prenatal—fetoscopic	5 (7.04%)
Haq et al., 2012 [15]	25	postnatal	0
Januschek et al., 2016 [16]	48	postnatal	0
Kahr et al., 2018 [17]	67	prenatal—open	1 (1.5%)
Kellogg et al., 2018 [18]	153	postnatal	5 (3.26%)
Kohn et al., 2018 [19]	34	prenatal—fetoscopic	n.s.
Lee et al., 2012 [20]	11	prenatal—n.s.	n.s.
Macedo et al., 2015 [21]	19	prenatal—n.s.	n.s.
Masini et al., 2019 [22]	157	postnatal	3 (1.91%)
Mohrlen et al., 2019 [23]	20	prenatal—open	1 (5%)
Moldenhauer et al., 2015 [24]	100	prenatal—open	6 (6.12%)
Moron et al., 2018 [25]	237	prenatal—open	5 (2.1%)
Musluman et al., 2012 [26]	162	postnatal	n.s.
Pastuszka et al., 2018 [27]	36	prenatal—open	n.s.
	36	postnatal	n.s.
Pedreira et al., 2016 [28]	10	prenatal—fetoscopic	2 (20%)
Protzenko et al., 2019 [29]	231	postnatal	n.s.
Sileo et al., 2019 [30]	67	postnatal	n.s.
Carraba et al., 2019 [33]	5	prenatal—fetoscopic	1 (20%)
Lapa et al., 2021 [34]	170	prenatal—fetoscopic	n.s.
Weaver et al., 2021 [32]	163	postnatal	n.s.
Spoor et al., 2019 [31]	93	postnatal	2 (2.15%)

n.s.—not stated.

**Table 7:** Comparison between surgical techniques.

Author, Year of Publication	Approach	Untethering	Dural Closure	Musculofascial Closure	Skin Closure
Haq et al., 2012 [15]	postnatal	7.0 interrupted suture	complete	complete	5.0 Monocryl
Musluman et al., 2012 [26]	postnatal	n.s.	71 cases—dural reconstruct	71 cases—fascia closure	*
Faria et al., 2013 [10]	prenatal—open	n.s.	n.s.	n.s.	n.s.
Macedo et al., 2015 [21]	prenatal—n.s.	n.s.	n.s.	n.s.	n.s.
Moldenhauer et al., 2015 [24]	prenatal—open	complete	primary running suture	running suture	running suture
Cherian et al., 2016 [11]	postnatal	n.s.	n.s.	n.s.	n.s.
Graf et al., 2016 [14]	prenatal—fetoscopic	n.s.	n.s.	n.s.	n.s.
Januschek et al., 2016 [16]	postnatal	complete	complete	n.s.	complete
Pedreira et al., 2016 [28]	prenatal—fetoscopic	n.s.	none	cellulose patch	running suture
Beuriat et al., 2017 [9]	postnatal	6.0 nonresorbable suture	4.0 resorbable suture	n.s.	n.s.
Elbabaa et al., 2017 [12]	prenatal—open	complete	complete	n.s.	yes
Kahr et al., 2018 [17]	prenatal—open	n.s.	n.s.	n.s.	n.s.
Kellogg et al., 2018 [18]	postnatal	n.s.	n.s.	n.s.	n.s.
Kohn et al., 2018 [19]	prenatal—fetoscopic	none	single layer skin-dura	none	single layer skin-dura
Moron et al., 2018 [25]	prenatal—open	complete	5.0 vycril	n.s.	5.0 Monocryl running suture
Pastuszka et al., 2018 [27]	prenatal—open	n.s.	n.s.	n.s.	n.s.
	postnatal	n.s.	n.s.	n.s.	n.s.
Beuriat et al., 2019 [8]	postnatal	n.s.	n.s.	n.s.	n.s.
Masini et al., 2019 [22]	postnatal	complete	n.s.	n.s.	n.s.
Mohrlen et al., 2019 [23]	prenatal—open	n.s.	n.s.	n.s.	n.s.
Protzenko et al., 2019 [29]	postnatal	n.s.	n.s.	n.s.	n.s.
Sileo et al., 2019 [30]	postnatal	n.s.	n.s.	n.s.	n.s.
Spoor et al., 2019 [31]	postnatal	complete	complete	musculofascial flap	primary suture/skin flap
Flanders et al., 2020 [13]	prenatal—open	n.s.	n.s.	n.s.	n.s.
	postnatal	n.s.	n.s.	n.s.	n.s.

* 43 cases—bilateral V-Y advancement flaps, 13 cases—Z plasty, 23 cases—bilateral bipedicle fasciocutaneous flaps, 10 cases—flap delaying procedure, n.s.—not stated.
